# Rasmussen’s encephalitis is characterized by relatively lower production of IFN-β and activated cytotoxic T cell upon herpes viruses infection

**DOI:** 10.1186/s12974-022-02379-0

**Published:** 2022-03-26

**Authors:** Yi-Song Wang, Dong Liu, Xin Wang, Qiao-Li Luo, Ling Ding, Dong-Ying Fan, Qi-Liang Cai, Chong-Yang Tang, Wei Yang, Yu-Guang Guan, Tian-Fu Li, Pei-Gang Wang, Guo-Ming Luan, Jing An

**Affiliations:** 1 Department of Microbiology, School of Basic Medical Sciences, Capital Medical University, Beijing, 100069 China; 2 Department of Neurosurgery, Sanbo Brain Hospital, Capital Medical University, Beijing, 100093 China; 3 Clinical Laboratory, Peking University International Hospital, Beijing, 102206 China; 4 MOE & NHC & CAMS Key Laboratory of Medical Molecular Virology, School of Basic Medical Science, Shanghai Medical College, Fudan University, Shanghai, 200032 People’s Republic Of China; 5 Center of Epilepsy, Beijing Institute for Brain Disorders, Beijing, 100093 China

**Keywords:** Rasmussen’s encephalitis, Herpes viruses, IFN-β, STING, IFI16

## Abstract

**Background:**

The etiology of Rasmussen's encephalitis (RE), a rare chronic neurological disorder characterized by CD8+ T cell infiltration and unihemispheric brain atrophy, is still unknown. Various human herpes viruses (HHVs) have been detected in RE brain, but their contribution to RE pathogenesis is unclear.

**Methods:**

HHVs infection and relevant immune response were compared among brain tissues from RE, temporal lobe epilepsy (TLE) and traumatic brain injury (TBI) patients. Viral antigen or genome, CD8+ T cells, microglia and innate immunity molecules were analyzed by immunohistochemical staining, DNA dot blot assay or immunofluorescence double staining. Cytokines were measured by multiplex flow cytometry. Cell apoptosis was visualized by TUNEL staining. Viral infection, immune response and the severity of unihemispheric atrophy were subjected to correlation analysis.

**Results:**

Antigens of various HHVs were prevalent in RE and TLE brains, and the cumulative viral score of HHVs positively correlated with the unihemispheric atrophy in RE patients. CD8+ T cells infiltration were observed in both RE and TLE brains and showed co-localization with HHV antigens, but their activation, as revealed by Granzyme B (GZMB) release and apoptosis, was found only in RE. In comparison to TLE, RE brain tissues contained higher level of inflammatory cytokines, but the interferon-β level, which was negatively correlated with cumulative viral score, was relatively lower. In line with this, the DNA sensor STING and IFI16, rather than other innate immunity signaling molecules, were insufficiently activated in RE.

**Conclusions:**

Compared with TBI, both RE and TLE had prevalently HHV infection and immune response in brain tissues. However, in comparison to TLE, RE showed insufficient activation of antiviral innate immunity but overactivation of cytotoxic T cells. Our results show the relatively lower level of antiviral innate immunity and overactivation of cytotoxic T cells in RE cases upon HHV infection, the overactivated T cells might be a compensate to the innate immunity but the causative evidence is lack in our study and need more investigation in the future.

**Supplementary Information:**

The online version contains supplementary material available at 10.1186/s12974-022-02379-0.

## Background

Rasmussen's encephalitis (RE) is a rare chronic neurological disorder mainly affecting children [1]. The clinical manifestations of RE are characterized by unilateral drug-resistant epilepsy, progressive neurological, cognitive deterioration, and brain atrophy [2, 3]. Hemispherectomy of the affected side still remains the only cure for controlling the seizures and cognitive deterioration caused by the disease [4–6].

Although much progress has been made in the pathogenesis of RE, the fundamental cause remains unknown. Viral infections and autoimmunity are considered to be possible causes. Since 1958 when RE was first reported [7, 8], several studies have reported the detection of a number of viruses including enteroviruses, Epstein-Barr virus (EBV), human cytomegalovirus (HCMV), human papillomavirus and herpes simplex virus (HSV) in the brains of RE patients. However, due to the variety of viruses detected, the lack of specificity, and the high infection rate of some viruses in the population, the causal relationship between a virus and RE has not yet been established.

In addition to viral infection, cytotoxic T lymphocyte (CTL) infiltration in the brain is a predominant feature of RE, and common T cell clones are shared among RE patients [9], indicating that the CTL-mediated immune response plays an important role in the pathogenesis of RE [10]. Autoimmunity was considered the cause of RE [11]. However, anti-inflammatory therapy showed poor efficacy, and autoantibodies were detected in only a few patients, suggesting that the precise antigen triggering the T-cell response still requires careful identification [12–15]. Recently, Anna et al. found that microglial nodules provides the environment for pathogenic T cells, accompanied with upregulation of interferon(IFN)-β [16]. This finding indicates a possible association between CTL and virus infection in RE pathogenesis, and suggests that the interaction between viral infection and the immune response may lead to RE disease.

Human herpes viruses (HHVs) consist of a group of double-stranded DNA viruses and include HSV1, HSV2, varicella zoster virus (VZV), EBV, HCMV, HHV6, HHV7 and Kaposi's sarcoma associated herpes virus (KSHV) [17]. Most of them are neurotropic and are likely to cause latent infection in the ganglion after acute infection [18, 19]. Previously, we detected antigens of HCMV, EBV and HHV6 with positive rates ranging from 50% to 88.5% of RE cases [20, 21]. The expression levels of HCMV, EBV and HHV6 antigens showed a positive correlation with brain atrophy, indicating the involvement of HHVs infection in this disease. However, HHV infection is common in humans and has a high seropositive rate in the population [17]. In addition to their presence in RE, HHV antigens have also been detected in other epilepsy seizure diseases, such as temporal lobe epilepsy (TLE) [22]. Therefore, the prevalence of HHV antigens in RE patients raises uncertainty about the effect of HHVs on pathogenesis of RE. Are HHVs true pathogenic factors or just an incidental coincidence?

To uncover the relationship between HHVs and RE, we collected more brain samples in this study, not only from RE patients, but also from TLE patients and from people suffering traumatic brain injuries (TBI). By comparing HHV antigen expression, CTL-mediated immune response and innate immune response in these samples, we found that RE was correlated with HHVs infection and characterized by relatively lower level of innate immune response and the release of GZMB by cytotoxic T cell.

## Materials and methods

### Patients

30 patients (16 males and 14 females) who met the 2005 European diagnostic criteria for RE [23] and underwent operation in Beijing Sanbo Brain Hospital from 2008 to 2018 were enrolled in this study. The scoring scheme of brain atrophy degrees of RE patients for the MRI was followed a previous report [24]. 20 patients (13 males and 7 females) who were diagnosed as temporal lobe epilepsy (TLE) and underwent operation due to intractable epilepsy, as well as 16 patients (10 males and 6 females) who underwent surgical treatment for traumatic brain injuries (TBI), were included as controls. RE patients had a mean age of seizure onset of 5.6 years and a mean age at surgery of 6.8 years. All TLE and TBI cases matched for age at the time of surgery.

### Brain tissue preparation

For RE and TLE cases, at least two blocks of brain tissues from different areas were collected. For TBI cases, brain tissue was collected at the edge of resected contusion area. The tissues were fixed, embedded, sliced, and then subjected to histopathological analysis. For the staining of each antigen, at least three sections from a single patient were used and each of the three sections was selected from ten successive sections so that the three sections were apart from each other at least 40 µm.

### Immunohistochemical staining (IHC)

In brief, the endogenous peroxidase activity and non-specific antibody binding sites were blocked with 3% hydrogen peroxide and 1% bovine serum albumin (BSA), respectively. After antigen retrieval, the sections were incubated at 4 °C overnight with the primary antibodies. The detailed information of the primary antibody was shown in Additional file 1. Then incubation with the secondary antibody and DAB was completed routinely. Counterstained with hematoxylin was used to visualize the outline of the cells. The counterstained nucleus served as the control, which is conducive to obtaining a clearer cell morphology. All sections were observed recorded, and photographed under a light microscope (Olympus X-cite 120, Olympus, Japan).

### Scoring methodology of IHC

The IHC results were scored as previously described [20]. Cells showing yellow or brown particles in the cytoplasm or nucleus were considered positive and were counted and analyzed using image analysis software (Image-Pro Plus 6.0; Media Cybernetics Inc., Bethesda, USA). The semi-quantitative results were expressed as the percentage of positive cells combined with a subjective assessment of staining intensity. The staining intensity was scored as 0 (colorless), 1 (light yellow), 2 (yellow or brown), and 3 (dark brown); the percentages of positive cells were denoted as 0 (< 5%), 1 (5–25%), 2 (26–50%), 3 (51%–75%), and 4 (> 75%). The multiplication of the score for staining intensity and that for percentages of positive cells was used to evaluate the immunostaining results as follows: overall scores of 0, 1− ≤ 3, > 3–6, and > 6 were defined as negative, weakly positive, moderately positive and strongly positive, respectively.

### Double immune-fluorescence staining

The sections were treated for antigen retrieval and then were simultaneously incubated with two primary or secondary antibodies. The information for all antibodies were shown in Additional file 1: Table S1. The images were taken using a laser scanning confocal microscope (TCS SP8 Leica, Germany).

### DNA Dot blot assay

Total DNA was extracted using a DNA extraction kit (DNeasy Blood & Tissue Kit, Qiagen, Germany), and DNA dot blot assay was conducted according to Cai’s protocol [25]. Briefly, denatured DNA was added to a nylon membrane to generate a dot array, and then DNA was fixed with UV-light for 7 kJ/10 min, followed by incubation with the preheat DIG Easy Hyb buffer in at 37 °C for 30 min. Denature DIG labeled DNA was treaded by boiling water and cooling in ice, and then was incubated with DIG Easy Hyb buffer at 42 °C overnight, and followed by washing with Blocking buffer. Then, after incubation with 5 mL DIG antibody solution at 25 °C for 1 h, Chemiluminescent substrate in alkaline phosphatase detection buffer was added to the membrane.

### TUNEL staining

The apoptosis was analyzed using an In Situ Cell Death Detection Kit, Fluorescein (11684795910, Roche) according to the manufacturer’s instructions (520 nm) and counterstained with DAPI (460 mm) for nuclei in paraffin sections. The images were taken using a confocal laser microscope (TCS SP8 Leica, Germany). Five none-overlapping views of lesion area in each section were chosen randomly by an investigator blinded to the experiment design.

### Multiplex flow cytometry

The cytokine levels in brain tissue and cerebrospinal fluid (CSF) samples of patients were quantified by AimPlex Human Multiple Immunoassays kits (Beijing Quantobio Biotechnology Co., China). Briefly, antigen was incubated with capture antibody conjugated beads, then with biotinylated detection antibodies, and finally with streptavidin-PE successively. Fluorescence signals of the sample were acquired by a flow cytometer (NovoCyte D1040) and levels of cytokines were analyzed with FCAP Array 3.0.

### Statistical analysis

For the scoring data obtained from IHC image, Mann–Whitney U test and Spearman's rank correlation coefficient was used to compare the difference and determine the correlation respectively. For analysis involving cumulative viral score or MRI score, Student’s t-test and Spearman's rank correlation coefficient was used to compare the difference and determine the correlation respectively. For data from multiplex flow cytometry, Student’s t-test and Pearson correlation coefficient was used to compare the difference and determine the correlation respectively. Statistical analysis was performed using SPSS 23.0 software. *P*-values < 0.05 were considered statistically significant.

## Results

### Antigens of various HHVs were detected in both RE and TLE brain tissues

**Fig. 1:**
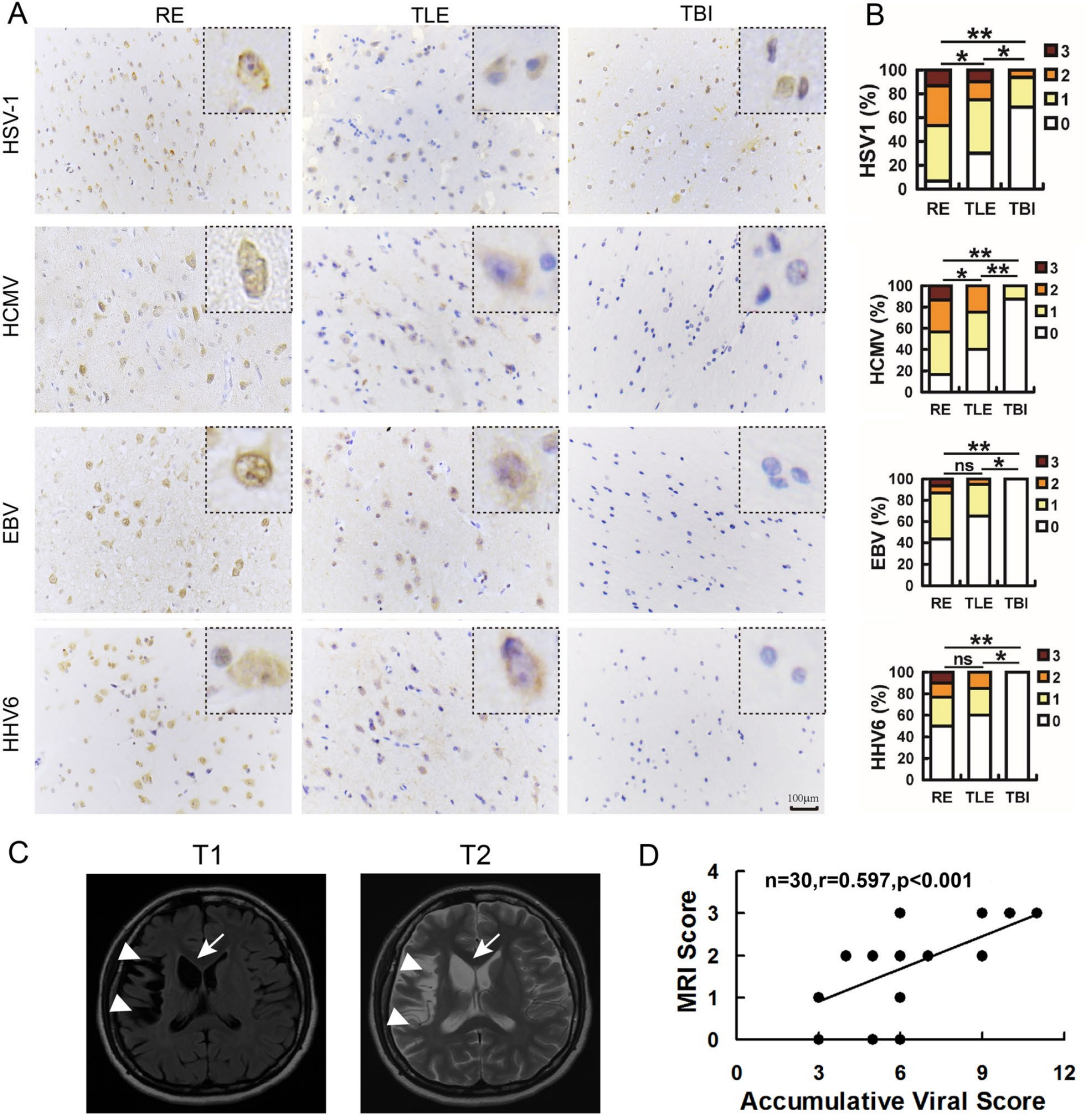
The detection of HHVs in RE and TLE brain samples and its correlation with MRI severity. **A** Sections from 30 RE, 20 TLE and 16 TBI brain samples was analyzed using immunohistochemistry (IHC) with antibodies against various HHVs and **B** were scored according to criteria described in the Material and Methods and analyzed using a two-tailed Mann–Whitney U test (*: *p* < 0.05; **: *p* < 0.01, ns: nonsignificance). Bar: 100 μm. **C** A representative magnetic resonance imaging (MRI) scan of an RE patient. The MRI scan shows a grey or white matter T2/FLAIR hyperintense signal and a hyperintense signal or atrophy of the cortex (arrowhead) and ipsilateral caudate head (arrow). The severity of RE was determined by grading brain atrophy detected by MRI and was expressed as an MRI score. The scoring scheme for the MRI was as following: 0 means no atrophy and no ventricle or sulcus enlargement, 1 means weak atrophy with the sulci deepening without ventricle enlargement, 2 means moderate atrophy and sulci deepening with moderate ventricular dilatation of less than half of the contralateral ventricular dilatation, and 3 means severe atrophy and sulci deepening with severe ventricular dilatation of more than half of the contralateral ventricular dilatation. **D** The correlation between cumulative viral scores and MRI scores was analyzed with Spearman's rank correlation coefficient

To address the association between HHV infection and RE, we collected 30 RE, 20 TLE and 16 TBI brain samples and antigen expression of eight HHVs was analyzed using IHC. Antigens of four HHVs including *HSV1, EBV, HCMV and HHV6* were detected (Fig. 1A and B). In TBI which represented brains from healthy people, the HHV-positive rate varied from 0 to 30%, and 40% had no antigen detected, indicating that HHV antigens positive rate in brains were not as common as the high seropositive rate in the population. In contrast, antigens of the four HHVs were universally detected in RE and TLE brains with different positive rates, which in both were significantly higher than those in TBI. Coinfection by more than one HHV were very common in RE and TLE samples. On average, each of them had 3 types of HHVs coinfected. In 18% of RE cases and 2% of TLE cases, all four HHV antigens were simultaneously detected (Additional file 1: Fig. S1A).

Among the four HHVs, HSV-1 and HCMV showed higher scores in RE than in TLE (Fig. 1A and B). Moreover, the cumulative viral score (cumulative score of coinfected HHVs in a single case) was significantly higher in RE than in TLE samples, suggesting that the HHV infection in RE was severer than that in TLE. Nevertheless, the difference between RE and TLE was much smaller than their difference from TBI (Additional file 1: Fig. S1B).

Dot blot hybridization was used to measure the content of HHV genomes in RE and TLE brain tissue. KSHV was used as a negative control. Among all HHVs, HSV1, EBV and HCMV were partially detected, with significantly higher signals in RE samples (Additional file 1: Fig. S2). The genome of HHV-6 was not detected in RE and TLE brain tissues, likely due to integration of HHV6 into the host telomeres in latency [26]

To validate the involvement of HHVs infection in RE, the association between the cumulative viral score and RE severity (grade of brain atrophy measured using magnetic resonance imaging (MRI) score) (Fig. 1C) was evaluated. It was found that the cumulative viral score positively correlated with unihemispheric atrophy (*r* = 0.597, *p* < 0.001, *n* = 30) (Fig. 1D), showing a close association between the progression of RE and HHV infection. However, no specific type of HHVs was exclusively detected in RE cases, and the difference between RE and TLE was much smaller than their difference from TBI. It is reasonable to believe that HHVs play an important role in RE progression but there must be other factors besides HHV infection that determine the occurrence of RE.

### CD8+ T cells infiltrated in the RE and TLE brain but were activated only in RE

**Fig. 2:**
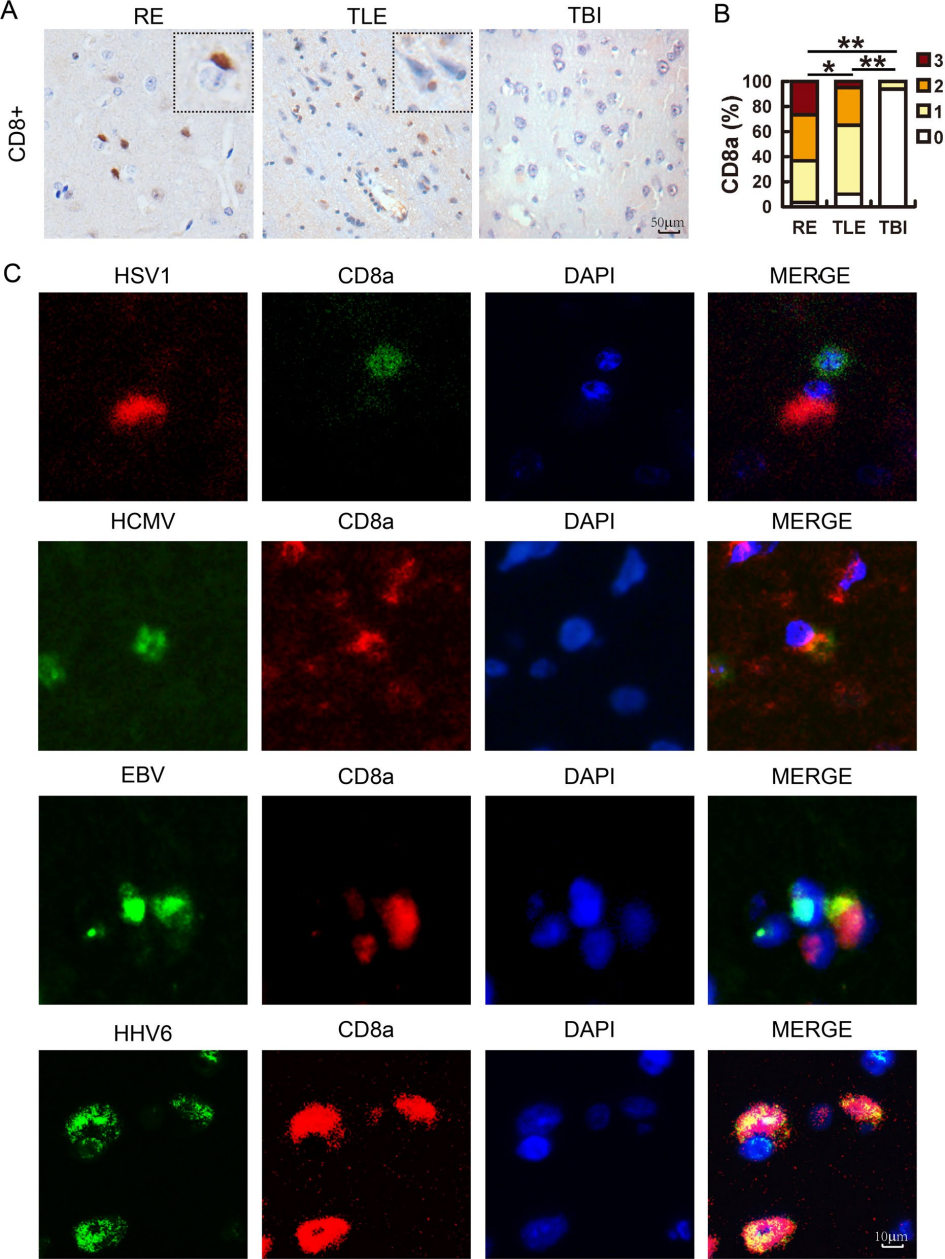
The detection of cytotoxic T cells in RE and TLE brain samples and its association with HHVs. **A** Sections from 30 RE, 20 TLE and 16 TBI brain samples were analyzed using IHC with antibodies against CD8a, and **B** were scored according to criteria described in the Materials and Methods and analyzed using a two-tailed Mann–Whitney U test (*: *p* < 0.05; **: *p* < 0.01). Bar: 50 μm. **C** Coimmunostaining with an antibody against CD8a and antibodies against various HHVs. Cell nuclei were visualized with DAPI. Bar: 10 μm

To identify factors beyond HHV infection, CD8+ T cell infiltration and the associated effector molecules were investigated in RE, TLE and TBI samples. IHC with anti-CD8a antibody showed positive staining in 96.7% of RE cases (Fig. 2A), indicating a wide presence of CD8+ T cells in the RE brain. Although 90% of TLE cases were immunoreactive to anti-CD8a antibody and the staining was much stronger than that in TBI, which had fewer CD8+ T cells detected, the signals were significantly weaker than those in RE cases (Fig. 2B).

The distribution of CD8+ T cells in RE brains has been well recognized, but its presence in TLE brains was unexpected. To investigate if the CD8+ T cells in RE and TLE brains were relevant to HHVs, the correlation analysis was performed between CD8a score and those of HHV antigens. As expected, the CD8a score was correlated with the cumulative viral score (*r* = 0.404, *p* = 0.027, *n* = 30) (Table 1). To validate whether the infiltrating CD8+ T cells targeted HHVs, coimmunostaining was performed in RE brains. For each kind of HHV antigen detected, CD8+ T cells were observed around the infected cells (Fig. 2C), indicating interaction between the HHV-infected cells and CD8+ T cells.

**Table 1:** Spearman's rank correlation coefficient among IHC score

	HSV1	HCMV	EBV	HHV6	CVS	Iba1	MRI
CVS	0.414*	0.412*	0.203	0.531**	1	0.277	0.597**
CD8a	0.078	0.177	0.215	0.087	0.404*	0.497**	0.285

The correlation among the IHC scores of RE samples was analyzed with Spearman's rank correlation coefficient (*n* = 30). CVS, cumulative viral score

^*^: *p* < 0.05; **: *p* < 0.01; *n* = 30

**Fig. 3:**
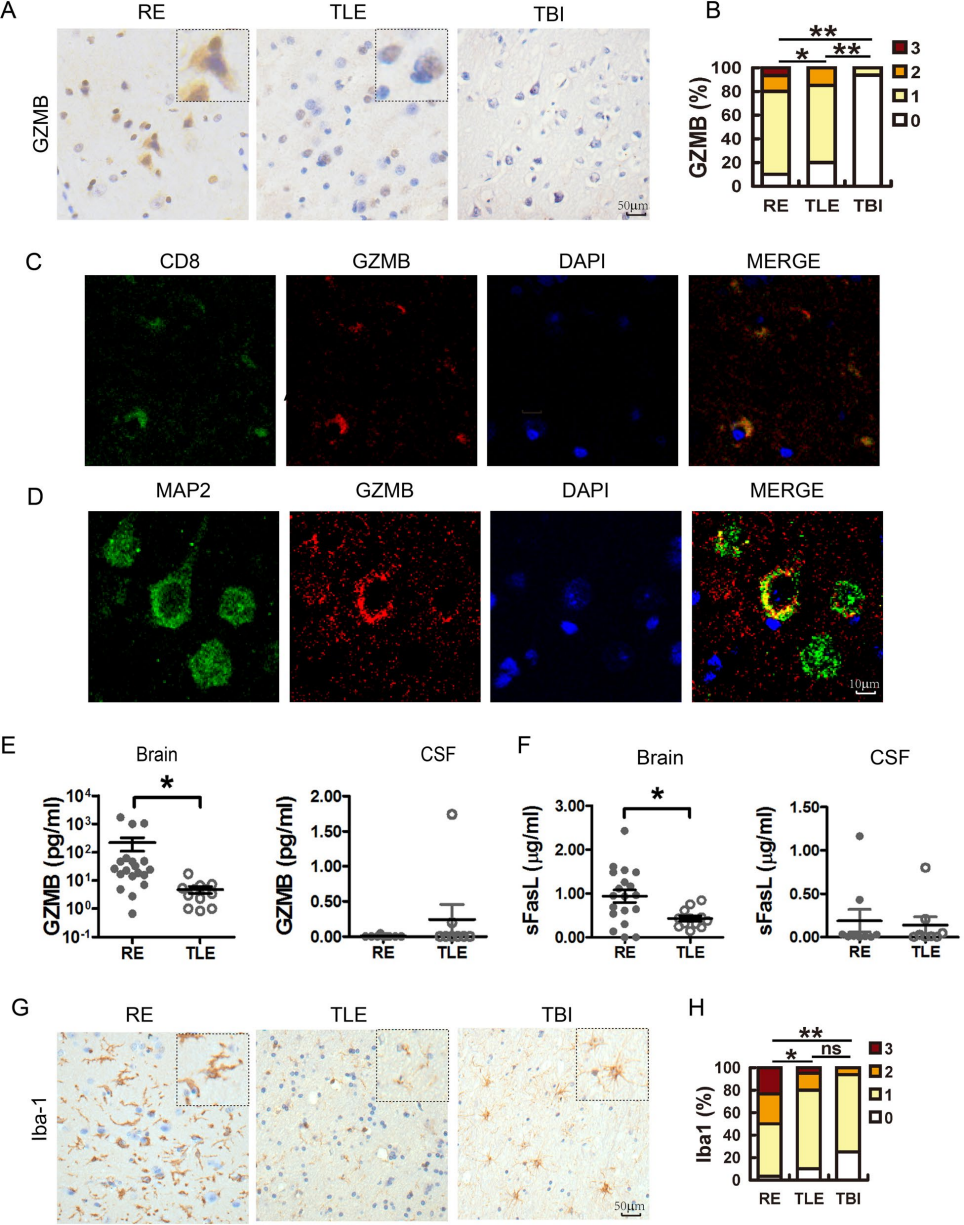
The detection of GZMB in RE and TLE brain and its colocalization with CD8a-positive cells and MAP2-positive cells. **A** Sections from 30 RE, 20 TLE and 16 TBI brain samples were analyzed using immunohistochemistry (IHC) with antibodies against GZMB, and **B** were scored according to criteria described in the Materials and Methods and analyzed using a two-tailed Mann–Whitney U test (*: *p* < 0.05; **: *p* < 0.01, ns: non-significance). Bar: 50 μm. **C** and **D** Coimmunostaining with antibody against GZMB and antibodies against CD8a (**C**) or MAP2 (**D**). Cell nuclei were visualized with DAPI. Bar: 10 μm. **E** and **F** The GZMB (**E**) and sFasL (**F**) levels in brain tissue (*n* = 19) and cerebrospinal fluid (CSF) samples (*n* = 12) of patients were quantified by AimPlex Human Multiplex Immunoassays, expressed as mean ± STD and analyzed using a two-tailed Student t test (*: *p* < 0.05). **G** and **H** The microglia were detected with antibodies against Iba1, scored and analyzed using a two-tailed Mann–Whitney U test. Bar: 50 μm

CD8+ T cells were present in both RE and TLE brains, while atrophy was only observed in RE. Moreover, the severity of RE (MRI score) was not correlated with CD8a score (*r* = 0.285, *p* = 0.127, *n* = 30) (Table 1). This inconsistency led us to analyze granzyme B (GZMB), the main effector molecule of CD8+ T cells. As revealed by IHC, only 6.3% of TBI cases were weakly stained while 90% of RE and 80% of TLE cases were positively reactive to anti-GZMB antibody (Fig. 3A and B), which colocalized well with CD8a (Fig. 3C). Moreover, in RE cases, neurons, which were the main target of HHVs (Additional file 1: Fig. S3) were sometimes stained with anti-GZMB antibody (Fig. 3D), providing direct evidence that neurons were attacked by CD8+ T cells through the production of GZMB. By AimPlex®Multiple Immunoassays, the GZMB levels in RE brain tissues were quantified and they ranged from 1000 pg/ml to 100 pg/ml in most cases, and was almost two logs higher than that in TLE tissues (Fig. 3E). A similar result was found for soluble FasL (sFasL), another effector molecule of CD8+ T cells (Fig. 3F). A possible explanation is that CD8+ T cells in TLE samples expressed but only release a small amount of GZMB, while those in RE released a lot of GZMB into the target cells, so the total amount of GZMB increased. These results thus implied that although TLE and RE cases were similarly infected with HHVs, CD8+ T cells in TLE cases were in a quiescent status, while those in RE cases were activated and released GZMB into HHV- infected cells.

### Microglial activation in RE cases was not correlated with the degree of atrophy

In addition to CD8+ T cell infiltration, the activation of microglia was investigated by scoring the presence of ionized calcium-binding adapter molecule 1 (Iba1), a marker of microglia. As shown in Fig. 3G and H, Iba1+ microglia with ameboid form were prevalent in RE cases and their score was significantly greater than those in TLE and TBI cases, indicating activation of microglia in RE. However, coimmunostaining revealed that microglia were not the target cell of HHV infection (Additional file 1: Fig. S4), and the Iba1 score was not correlated with MRI score (*r* = 0.140, *p* = 0.462, *n* = 30). However, Iba1 score was correlated with CD8a score (*r* = 0.497, *p* = 0.005, *n* = 30) (Table 1), and apoptosis was more obvious in RE than in TLE or TBI samples (Additional file 1: Fig. S5), indicating that the microglia were activated by apoptosis induced by CD8+ T cells. The association between microglia and CD8+ T cells was also supported by the amoeboid form of microglia in RE cases, which is usually observed when those cells are clearing extracellular debris and apoptotic cells [27].

### The RE brain produced more T cell relevant cytokines

**Fig. 4:**
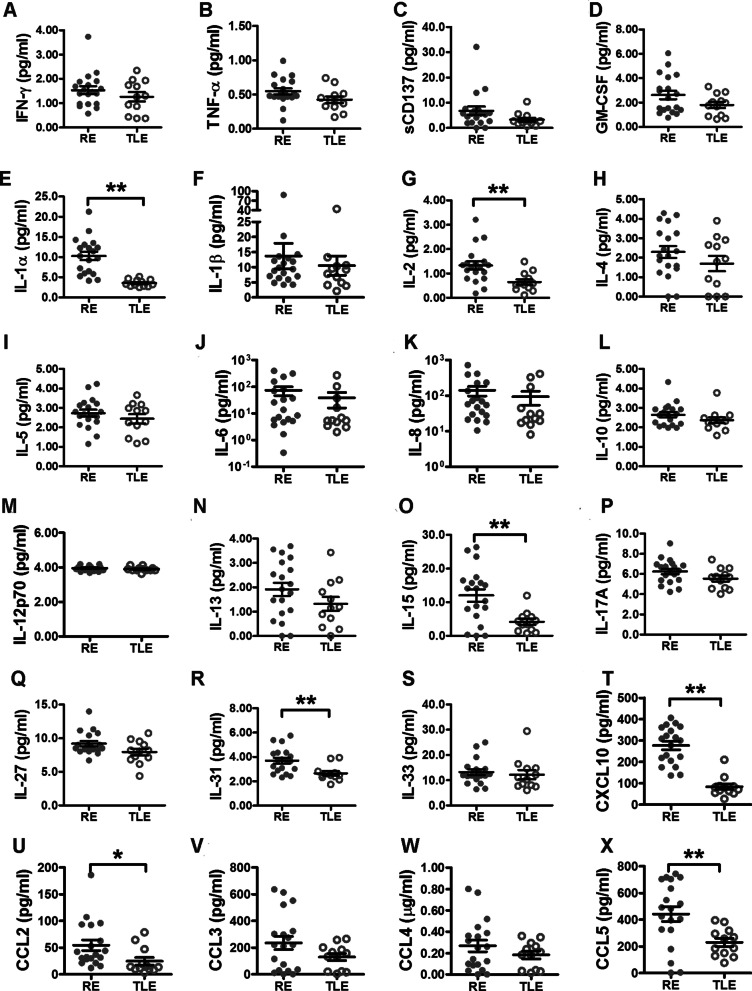
Cytokines levels in RE and TLE brain tissue samples. Brain tissues from RE and TLE patients were analyzed using AimPlex Human Multiplex Immunoassays kits according to the manufacturer’s instructions. The expression levels of IFN-γ (**A**), TNF-α (**B**), sCD137 (**C**), GM-CSF (**D**), IL-1α (**E**), IL-1β (**F**), IL-2 (**G**), IL-4 (**H**), IL-5 (**I**), IL-6 (**J**), IL-8 (**K**), IL-10 (**L**), IL-12p70 (**M**), IL-13 (**N**), IL-15 (**O**), IL-17A (**P**), IL-27 (**Q**), IL-31 (**R**), IL-33 (**S**), CXCL10 (**T**), CCL2 (**U**), CCL3 (**V**), CCL4 (**W**), CCL5 (**X**), were expressed as the mean ± std and analyzed with a two-tailed Student t test (*n* = 19 for RE and *n* = 12 for TLE). *: *p* < 0.05; **: *p* < 0.01

To determine the mechanisms underlying the activation of CD8+ T cells in RE cases in the presence of an HHV infection, a panel of inflammatory cytokines in the brain tissue and cerebrospinal fluid (CSF) of RE and TLE patients were measured. As shown in Fig. 4, most proinflammatory and anti-inflammatory cytokines, such as interferon-γ (IFN-γ), tumor necrosis factor alpha (TNF-α), interleukin-1β (IL-1β), IL-6, IL-4, and IL-10, didn’t show significant difference between RE and TLE brain tissues (Fig. 4A–S). The exception was IL-1α, which is usually released by damaged cells [28] and markedly increased in RE samples (Fig. 4E).

Among all cytokines tested, the most significant difference between RE and TLE cases was observed for IL-2 (Fig. 4G), IL-15 (Fig. 4O), C–X–C motif chemokine 10 (CXCL10) (Fig. 4T) and C–C motif chemokine ligand 5 (CCL5) (Fig. 4X). Cytokines in CSF displayed a similar trend as those in brain tissue, but only IL-1α and CXCL10 showed significantly increases when compared with those in TLE (Additional file 1: Fig. S6). The CXCL10/CXCR3 signaling pathway and IL-15 play indispensable roles in recruiting and activating HSV-1 specific CD8+ T cells in trigeminal ganglia with HSV-1 latency infection [29, 30]. The increase in these two cytokines hence provided further evidence for the activation of CD8+ T cells in RE cases.

### Interferon-β was produced in RE brain tissues at a relatively lower level

**Fig. 5:**
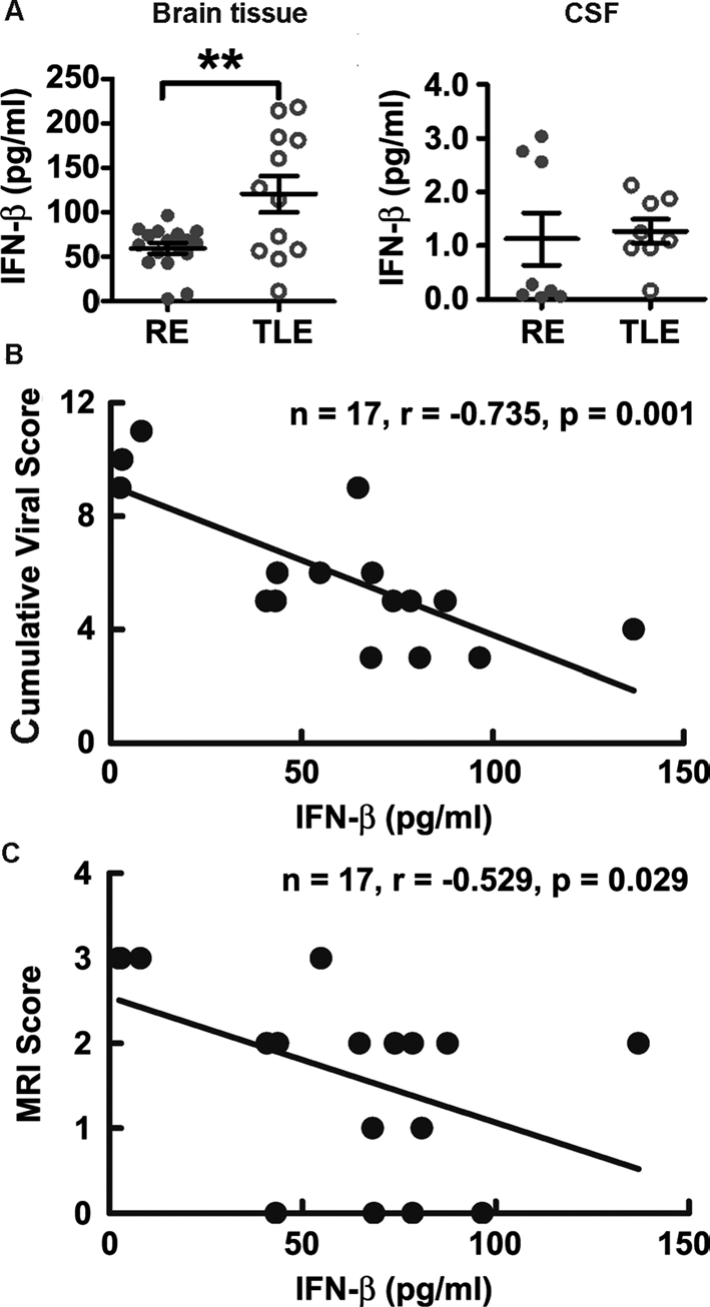
The levels of IFN-β in RE and TLE brain tissues and their correlation with viral infection and severity in RE cases. **A** IFN-β in brain tissue or CSF from RE and TLE patients were measured using AimPlex Human Multiple Immunoassays kits, expressed as the mean ± std and analyzed with a two-tailed Student’s t test (*n* = 19 for RE and *n* = 12 for TLE in brain tissue measurement; *n* = 9 for RE and *n* = 8 for TLE in CSF measurement). **: *p* < 0.01. **B** and **C** The correlation between (c) cumulative viral score or (d) MRI score and IFN-β level in brain tissue was analyzed with Spearman's rank correlation coefficient (n = 17)

To understand why CD8+ T cells were only activated in RE cases, antiviral innate immunity was characterized. As the key factors in antiviral innate immunity, IFN-β was detected in brain tissues of both RE and TLE cases, but its level was significantly lower in RE cases (Fig. 5A). The results indicated that, although produced IFN-β in response to HHV infection, the antiviral innate immunity was not fully activated in RE cases.

Correlation analysis was further performed for all chemokines with significant changes in RE cases including GZMB and sFasL (Table 2). IL-2, IL-15 and CCL5 were positively correlated with GZMB and/or sFasL, validating their roles in recruiting and activating CD8+ T cells. The positive correlation between IL-1α and GZMB might indicate that activated CD8+ T cells released a large amount of GZMB that damaged neurons, which may be the main source of increased IL-1α. Intriguingly, IFN-β was negatively correlated with all other chemokines. Furthermore, the IFN-β level in brain tissue was negatively correlated with both the viral cumulative score and MRI score (Fig. 5B and C), suggesting that insufficient production of IFN-β could not control the HHV infection and promoted the development of brain atrophy.

**Table 2:** Pearson’s correlation coefficient among significantly changed cytokines

	IL-1α	IL-2	IL-15	CCL5	CXCL10	GZMB	sFasL	IFN-β
IL-1α	1							
IL-2	0.419	1						
IL-15	0.312	0.695**	1					
CCL5	0.323	0.726**	0.934**	1				
CXCL10	0.855**	0.396	0.402	0.422	1			
GZMB	0.540*	0.449	0.536*	0.481**	0.377	1		
sFasL	0.308	0.633**	0.951**	0.932**	0.393	0.645**	1	
IFN-β	− 0.425	− 0.373	− 0.276	− 0.319	− 0.671**	− 0.207	− 0.248	1

The correlation among cytokines that showed significant differences between RE and TLE cases was analyzed with Pearson correlation (*n* = 17 for IFN-β and *n* = 19 for the others)

^*^: *p* < 0.05; **: *p* < 0.01. *N* = 17 for IFN-β and *n* = 19 for others

### The staining signal of STING and IFI16 were lower in the RE brain

**Fig. 6:**
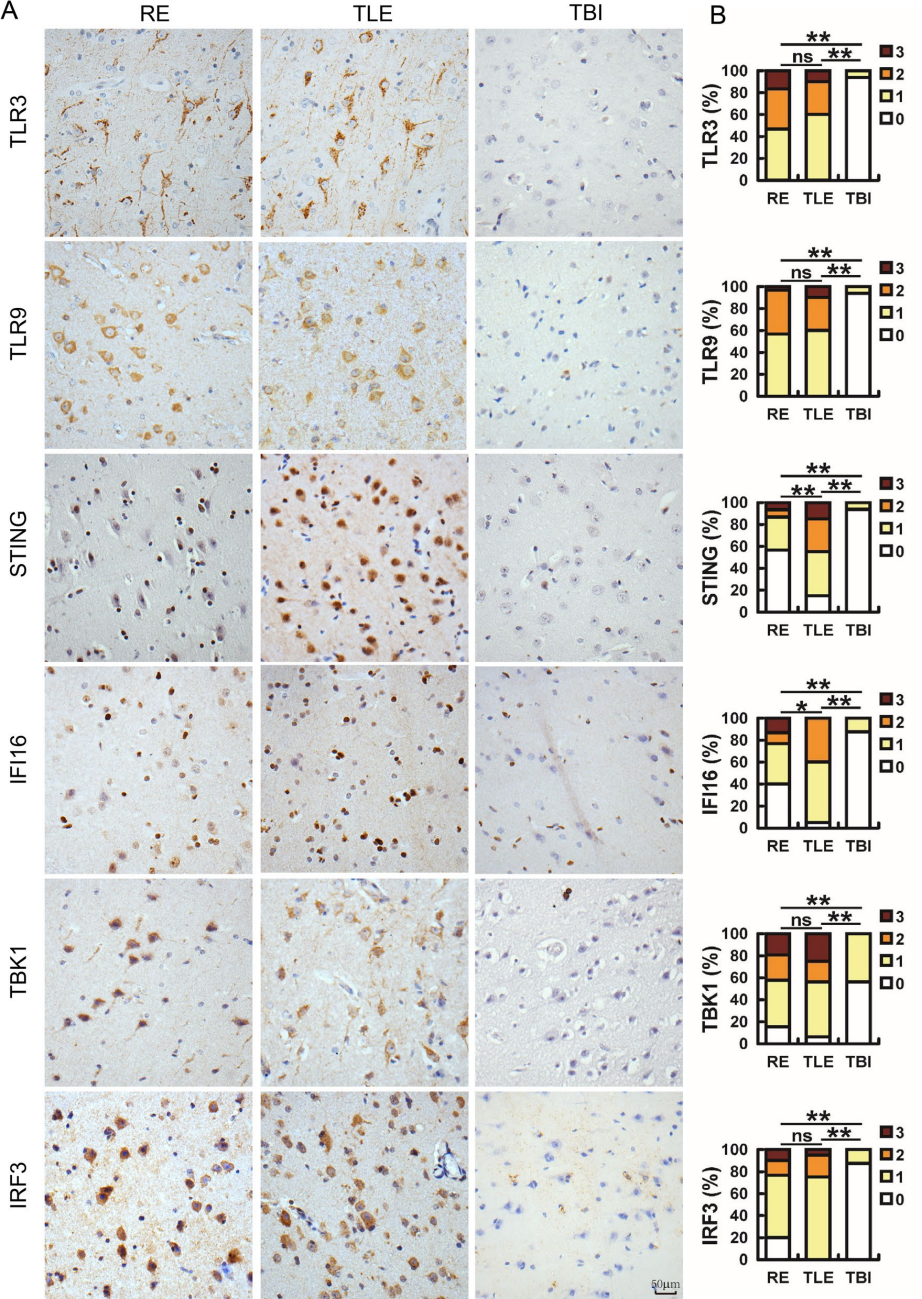
The detection of innate immunity factors in RE and TLE brain samples. **A** Sections from 30 RE, 20 TLE and 16 TBI brain samples were analyzed using immunohistochemistry (IHC) with antibodies against TLR3, TLR9, STING, IFI16, TBK1 or IRF3, and **B** were scored according to criteria described in the Materials and Methods and analyzed using a two-tailed Mann–Whitney U test (*: *p* < 0.05; **: *p* < 0.01, ns: nonsignificance). Bar: 50 μm

Because IFN-β was produced relatively lower in RE brains, the activation of signaling pathways involved in antiviral innate immunity was investigated by grading the expression of key DNA sensors and their downstream factors. Toll-like receptor 3 (TLR3), TLR9, stimulator of interferon genes (STING), and γ-interferon-inducible protein 16 (IFI16) recognize DNA in the cytoplasm, cytoplasm/nucleus and nucleus, respectively. Compared with those in TBI patients, the scores of these molecules and their downstream factors such as serine/threonine protein kinase (TBK1) and interferon regulatory factor 3 (IRF3) were significantly increased in RE and TLE patients (Fig. 6A and B), consistent with the increase in HHV antigens.

Between RE and TLE tissue sections, no obvious change was observed either in the distribution or signal intensity of TLR3, TLR9, TBK1 and IRF3. However, the STING and IFI16 scores were significantly lower in RE sections, especially for STING whose positive rates were 43.4% and 85% in RE and TLE, respectively (Fig. 6A and B), which may explain insufficient IFN-β levels in RE cases because the activation of STING and IFI16 have been shown to stimulate the production of IFN-β [31]. Those results indicated that STING and IFI16 signaling in brain of RE patients was disrupted when in the presence of HHV infection and provided further evidence showing that anti-viral innate immunity was not activated sufficiently.

## Discussion

Recently, increasing evidence has revealed the crucial role of the immune process in RE pathogenesis, but the primary cause that initiates immunity remains to be elucidated. In this study, we demonstrated that HHV antigens were prevalent in both RE and TLE patients. However, these two diseases displayed distinct patterns with respect to the status of infiltrated cytotoxic T cells, the production of cytokines, the polarization of microglia and the activation of DNA sensors, indicating that different immune responses to HHV infection occurred in RE and TLE cases. In particular, the STING and IFI16 at protein level, as well as the production of IFN-β were lower in RE patients, but the activation of CD8+ T lymphocytes was stronger. This implied that the innate immune response in RE cases was relatively less activated and that the compensating activated cytotoxic T cells inclined to eliminate HHV-infected neural cells. In contrast, a balance between host immunity and HHVs maintained a steady state in the brains of TLE cases. Thus, the brains of RE was continually damaged and developed into a progressive neurological disorder characterized by brain atrophy.

Viral infection has long been proposed as the etiology of RE [7, 8]. In our previous research, antigens of various HHVs including HCMV, EBV and HHV6 were detected in RE brain tissues [20, 21]. Nevertheless, HHV infection is very common in humans [17], so that the prevalence of HHV antigens in RE patients did not necessarily mean that HHVs were pathogenic factors. In the current work, by comparing viral antigens in RE, TLE and TBI samples, we found that HHV antigens were dominant in RE and TLE brains but not in TBI cases. On the one hand, the presence of HHVs in both RE and TLE samples indicated that HHVs were not specifically associated with RE, so that HHV infection would not directly lead to RE. On the other hand, the detection of HHVs in RE and TLE were much more common than in TBI brains so that their presence in some situations was likely to trigger the pathogeneses of brain disorders such as RE and TLE.

Notably, the observation of herpes viruses in RE patients’ brains does not mean the identification of a specific pathogen for the development of RE. In our research, more than one type of herpes virus was found in RE brain, and they were all able to trigger an abnormal immune response in RE patients. Based on these finding, we hypothesized that RE was caused by an imbalanced immune response triggered by some pathogen infections, rather than a specific pathogen. Moreover, it cannot be excluded that viruses other than herpes virus may perform similar effect as herpes virus although we had no direct evidence yet.

The mechanisms underlying the prevalence of herpes viruses in brains of RE patients are unknown. Some herpes viruses usually cause infection in unilateral body such as cold sores, genital herpes and herpes zoster caused by HSV-1, HSV2 and VZV infections, respectively. In general, after the primary infection, herpes viruses are transported along the nerve fibers into brain and persist there. When resistance of the host is low, the latent herpes viruses are activated and cause infection. The repeated latency and activation may link to RE pathogenesis that often occurs in unihemisphere. Although most herpes viruses are neurotropism virus and prevalent in population, their presence in health people is much lower than RE patients and TLE patients. The abnormalities in immune response, somatic mutations, somatic mosaicism, and the up regulation of regulators of T-cell function in chronic viral infections might be the possible factors for the susceptibility of RE patients to herpes virus infection. The above questions deserved to further investigation in the future.

Besides HHV infection, we also investigated the infiltration of CD8+ T cells in the brain. Although their role in the development of RE is supported by a number of studies [31] as well as our own results, the antigen triggering the CD8+ T-cell response is still unknown. According to our results, both RE and TLE patients had CD8+ T cell infiltration in the brain, indicating that CD8+ T cells were likely to be recruited by HHV infection. Moreover, our study showed that neurons were the dominant cell types infected by HHVs, the infiltrated CD8+ T cells in the RE brain were often closely adjacent to HHV antigen-positive neuronal cells and neurons were co-stained with anti-GZMB antibody. These results suggest that CD8+ T cells primarily targeted HHV-infected neurons and that the infiltration of CD8+ T cells was the consequence of HHV infection. It has been demonstrated that HSV-1-specific memory CD8+ T cells are selectively activated and retained in sensory ganglia to block HSV-1 reactivation from latency [32, 33]. As latent HHV infection is difficult to eliminate, the CD8+ T cell-mediated immune response will coexist with the latent infection for a long time and thus lead to chronic but persistent damage to the brain, which might directly promote the occurrence and progression of RE. Furthermore, because all RE patients were infected with more than one type of HHV, the CD8+ T cells in the brain were possibly a divergent population that further deteriorated the immune-mediated injury. It must also be mentioned that a substantial number of CD8+ T cells appeared in the TLE brain, but their status seemed to be different. The coexistence of HHVs and quiescent CD8+ T cells has been reported in sensory ganglia, where HHV-specific CD8+ T cells are retained to prohibit HHV reactivation from latency [33]. Based on this observation, a possible explanation was that CD8+ T cells in RE cases were activated to kill the infected cells, while those in TLE cases were just monitoring their target cells. Notably, this is just a hypothesis and needs more data to support in the future.

The activation of CD8+ T cells in RE but not TLE cases was further supported by the cytokine analysis. During peripheral infection in ganglia, the CXCL10/CXCR3 signaling pathway is indispensable for recruitment of HHV-specific CD8+ T cells [29] and IL-15 plays an essential role in selectively activating these cells [30]. It has been demonstrated that reactivation of HSV-1 by UV-B significantly increases both the number and function of HSV-specific memory CXCR3+ CD8+ T cells in trigeminal ganglia latently infected with HSV-1. Moreover, the delivery of exogenous CXCL10 boosts the number and function of HSV-1 specific CD8+ T cells and improves the protection against recurrent herpesvirus infection and disease in mice [29]. In another experiment, incubation with IL-15 induced CD38/HLA-DR expression in HCMV- and EBV-specific CD8+ T cells while other inflammatory cytokines such as IL-2, IL-7, IFN-γ, IFN-α and TNF-α did not activate EBV and HCMV specific CD8+ T cells [34]. In our study, CXCL10 and IL-15 in brain tissues were the most significantly upregulated in RE than in TLE cases, consistent with the results that the infiltration of CD8+ T cells was prevalent in RE cases. All these results provided more evidence supporting the hypothesis that CD8+ T cells were activated in RE patients.

What caught our attention was the different clinical outcome between RE and TLE cases despite the large number of HHVs similarly detected in them. In subsequent experiments, we compared the innate immune response against HHVs between RE and TLE cases. During the infection process, HHV DNA can be recognized by DNA sensors TLR3/9, STING and IFI16, which are present in the endosome, cytoplasm and nucleus, respectively. STING and IFI16 activation has been shown to stimulate the production of type I interferon [14]. Moreover, IFI16 is also crucial for the latent infection of HHVs. Megan Orzalli reported that HSV-1 infection is sensed by nuclear IFI16 [35], and Gina further proved that IFI16 is required for the maintenance of EBV latency in vitro [36]. In our study, lower STING and IFI16 protein levels were observed in RE than in TLE cases, which consequently led to the insufficient production of IFN-β. IFN-β is a crucial cytokine of innate immunity and plays a key role in antiviral immunity. The insufficient IFN-β production would undoubtedly impair the defense against HHV infection, and make the HHVs easier to reactivate. This could explain why HHV antigens and genomes were slightly higher in RE samples. The mechanisms leading to the reduced expression of STING and IFI16 are not clear. An in-depth investigation at the level of biochemistry and genetics is required in the future.

## Conclusion

Based on these results, we propose the following model to explain the pathogenesis of RE. In people who suffer latent HHV infection in the CNS, innate immunity (IFN-β) and adaptive immunity (CD8+ T cells), collaborate to restrict the reactivation of HHVs. The balance between immunity and HHV latency cannot protect CNS in patients with functional disorders such as TLE, but does avoid the neuronal damage and loss. In RE cases, however, IFN-β production is not sufficient because of the lack of STING and IFI16 activation for some unknown reasons. Relatively lower lever of IFN-β production impairs the defense against HHVs and makes them easier to reactivate. As a result, the brain tissue increased the production of CXCL10 and IL-15 and consequently activated the HHV-specific CD8+ T cells. HHVs usually cause infection in the unilateral body, which may be linked to that RE pathogenesis also often occurs in unihemisphere. The activation of CD8+ T cells compensates for the insufficiently activated IFN-β and controls the spread of HHVs by inducing apoptosis in infected cells; however, it inevitably results in the loss of large number of neurons and finally leads to chronic unihemispheric brain atrophy. Although the proposed model explains many features of RE, it still needs further verification by in vitro studies and animal experiments. Moreover, the mechanisms leading to the insufficient expression of STING and IFI16 also need further investigation. Genetic variation of the host or mutation of the viruses are potential factors for the alternative signaling activation in RE. Although the detailed investigation into these possibilities is beyond the scope of this work, we acknowledge that they are important to ultimately understand the occurrence of RE, and efforts are currently underway in our lab to identify such factors.

## Supplementary Information

**Additional file 1: Fig. S1.** The coinfection of HHVs in RE and TLE. (a) Coinfection by more than one HHVs in RE, TLE and TBI cases. (b)The cumulative viral scores, which were the cumulative scores of coinfected HHVs in a single case, were expressed as mean ± STD and analyzed with a two-tailed Student t test(**: p < 0.01). **Fig. S2.** DNA dot hybridization for detecting genomes of HHVs in brain tissue. DNA from 15 RE and 10 TLE cases were transferred to a nylon membrane. Viral genomes were measured with DIG labeled probes targeting to various HHVs and visualized with DIG antibody. KSHV was used as a negative control Fig. 3**. (a)** Coimmunostaining with antibody against neuron marker microtubule-associated protein(MAP) 2 and antibodies for various HHVs. Cell nuclei were visualized with DAPI. Bar: 10 μm. **(b)** Standard curve of Granzyme and sFasL in AimPlex Human Multiplex Immunoassay. **Fig. S4.** The colocation of microglia with antigens of HHVs in RE and TLE brain tissues. Coimmunostaining with antibody against Iba1 and antibodies against various HHVs. Cell nuclei were visualized with DAPI. Bar: 50 μm. **Fig. S5.** Apoptosis from representative RE, TLE and TBI brain samples was analyzed using an In Situ Cell Death Detection Kit, and visualized by fluorescence method. Bar: 50 μm. **Fig. S6.** The cytokine levels in RE and TLE cerebrospinal fluid (CSF). CSF from RE and TLE patients were analyzed using Aimplex Human Multiplex Immunoassays kits according to the manufacturer’s instruction. The cytokine levels were expressed as mean ± std and analyzed with a two-tailed Student ttest (n = 9 for RE and n = 8for TLE). *: p < 0.05. **Table S1.** List of antibodies.

## Data Availability

Data supporting the conclusions of this article are presented in this manuscript.

## Abbreviations

RE: Rasmussen's encephalitis
HHV: Human herpes viruses
TLE: Temporal lobe epilepsy
EBV: Epstein-Barr virus
HCMV: Human cytomegalovirus
HSV: Herpes simplex virus
CTL: Cytotoxic T lymphocyte
VZV: Varicella zoster virus
KSHV: Kaposi's sarcoma associated herpes virus
TBI: Traumatic brain injuries
IHC: Immunohistochemical staining
MRI: Magnetic Resonance Imaging
