# Supplementary material for: Rasmussen’s encephalitis is characterized by relatively lower production of IFN-β and activated cytotoxic T cell upon herpes viruses infection
Source: J Neuroinflammation. 2022 Mar 26;19:70. doi: 10.1186/s12974-022-02379-0 (PMC8957159; doi:10.1186/s12974-022-02379-0)
Supplement: Supplementary file 1 — Additional file 1: Fig. S1. The coinfection of HHVs in RE and TLE. (a) Coinfection by more than one HHVs in RE, TLE and TBI cases. (b)The cumulative viral scores, which were the cumulative scores of coinfected HHVs in a single case, were expressed as mean ± STD and analyzed with a two-tailed Student t test(**: p < 0.01). Fig. S2. DNA dot hybridization for detecting genomes of HHVs in brain tissue. DNA from 15 RE and 10 TLE cases were transferred to a nylon membrane. Viral genomes were measured with DIG labeled probes targeting to various HHVs and visualized with DIG antibody. KSHV was used as a negative control Fig. 3. (a) Coimmunostaining with antibody against neuron marker microtubule-associated protein(MAP) 2 and antibodies for various HHVs. Cell nuclei were visualized with DAPI. Bar: 10 μm. (b) Standard curve of Granzyme and sFasL in AimPlex Human Multiplex Immunoassay. Fig. S4. The colocation of microglia with antigens of HHVs in RE and TLE brain tissues. Coimmunostaining with antibody against Iba1 and antibodies against various HHVs. Cell nuclei were visualized with DAPI. Bar: 50 μm. Fig. S5. Apoptosis from representative RE, TLE and TBI brain samples was analyzed using an In Situ Cell Death Detection Kit, and visualized by fluorescence method. Bar: 50 μm. Fig. S6. The cytokine levels in RE and TLE cerebrospinal fluid (CSF). CSF from RE and TLE patients were analyzed using Aimplex Human Multiplex Immunoassays kits according to the manufacturer’s instruction. The cytokine levels were expressed as mean ± std and analyzed with a two-tailed Student ttest (n = 9 for RE and n = 8for TLE). *: p < 0.05. Table S1. List of antibodies. [file 12974_2022_2379_MOESM1_ESM.pdf]

Supplementary Figure and Table

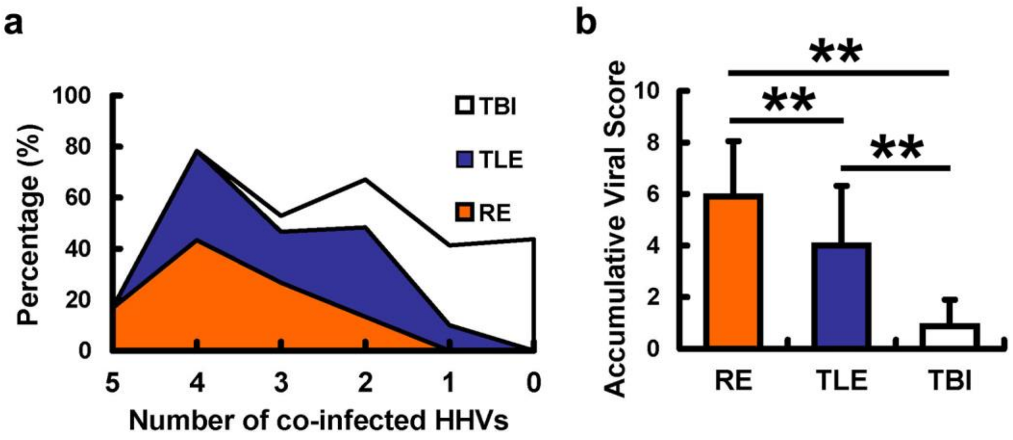

Supplementary Figure 1. The coinfection of HHVs in RE and TLE. (a) Coinfection by more than one HHVs in RE, TLE and TBI cases. (b) The cumulative viral scores, which were the cumulative scores of coinfected HHVs in a single case, were expressed as mean ± STD and analyzed with a two-tailed Student t test (**: p<0.01).

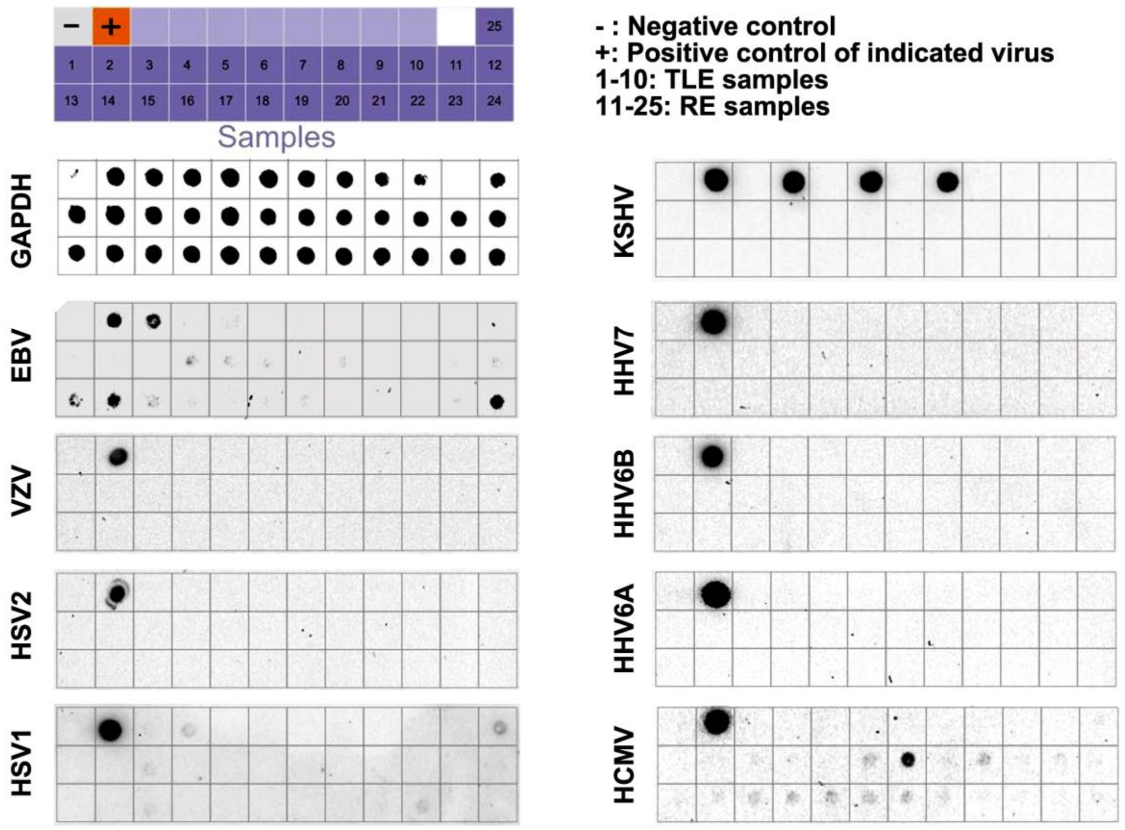

Supplementary Figure 2. DNA dot hybridization for detecting genomes of HHVs in brain tissue. DNA from 15 RE and 10 TLE cases were transferred to a nylon membrane. Viral genomes were measured with DIG labeled probes targeting to various HHVs and visualized with DIG antibody. KSHV was used as a negative control.

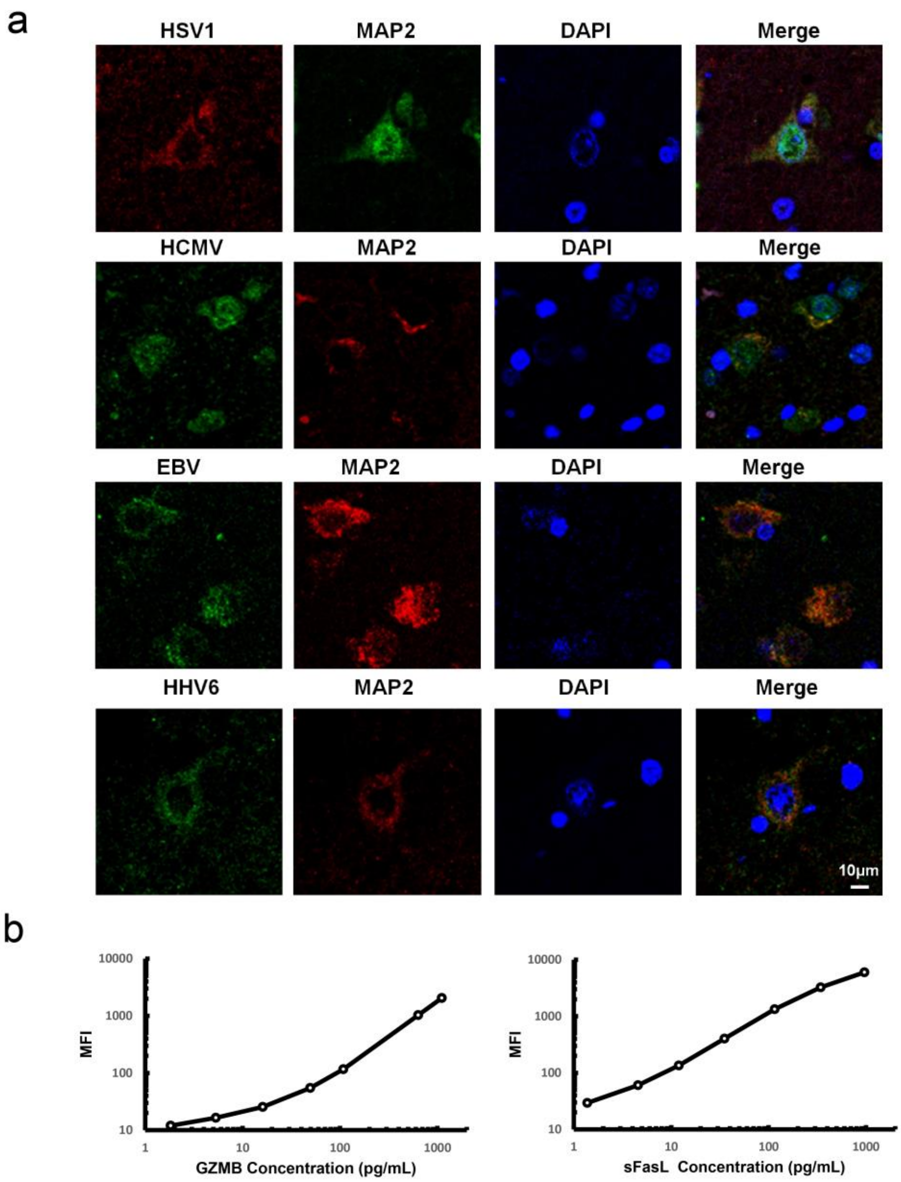

Supplementary Figure 3. (a) Coimmunostaining with antibody against neuron marker microtubule-associated protein (MAP) 2 and antibodies for various HHVs. Cell nuclei were visualized with DAPI. Bar: 10 μm. **(b)** Standard curve of Granzyme and sFasL in AimPlex Human Multiplex Immunoassay.

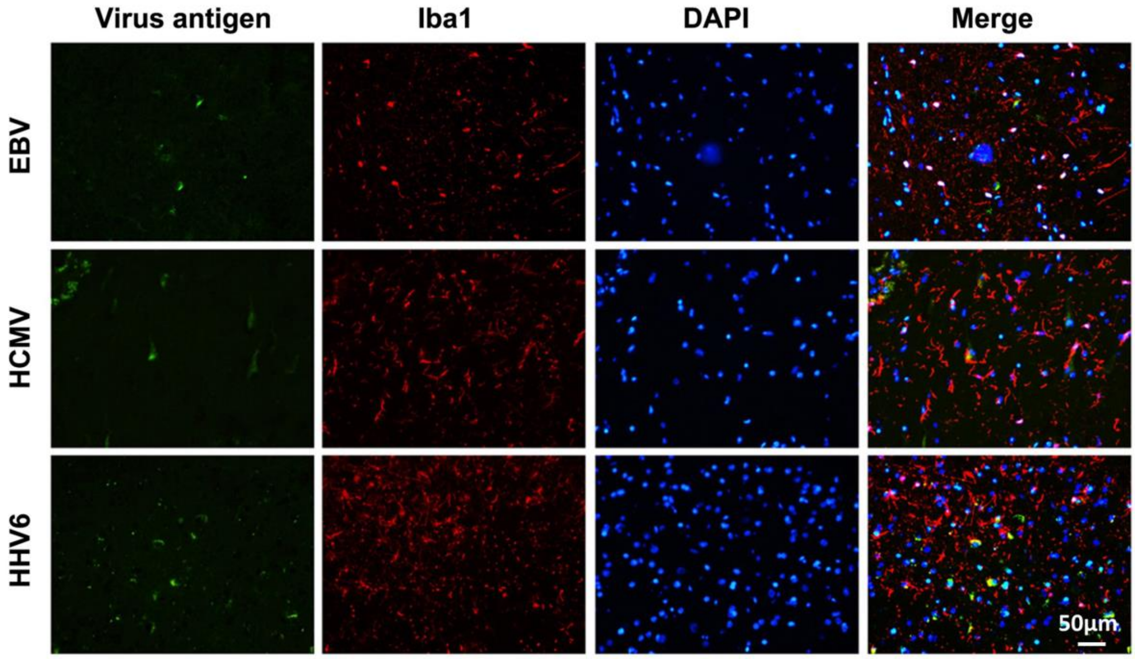

Supplementary Figure 4. The colocation of microglia with antigens of HHVs in RE and TLE brain tissues. Coimmunostaining with antibody against Iba1 and antibodies against various HHVs. Cell nuclei were visualized with DAPI. Bar: 50 μm.

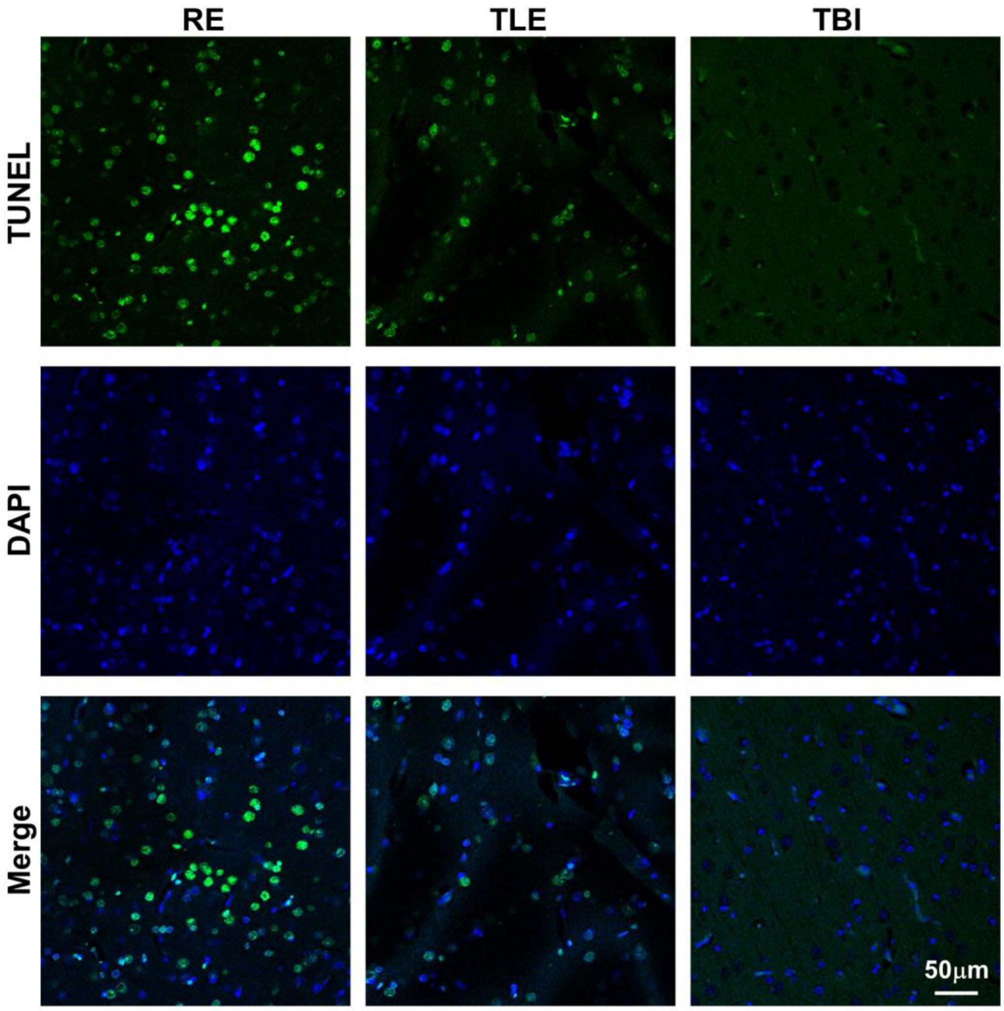

Supplementary Figure 5. Apoptosis from representative RE, TLE and TBI brain samples was analyzed using an In Situ Cell Death Detection Kit, and visualized by fluorescence method. Bar: 50 μm.

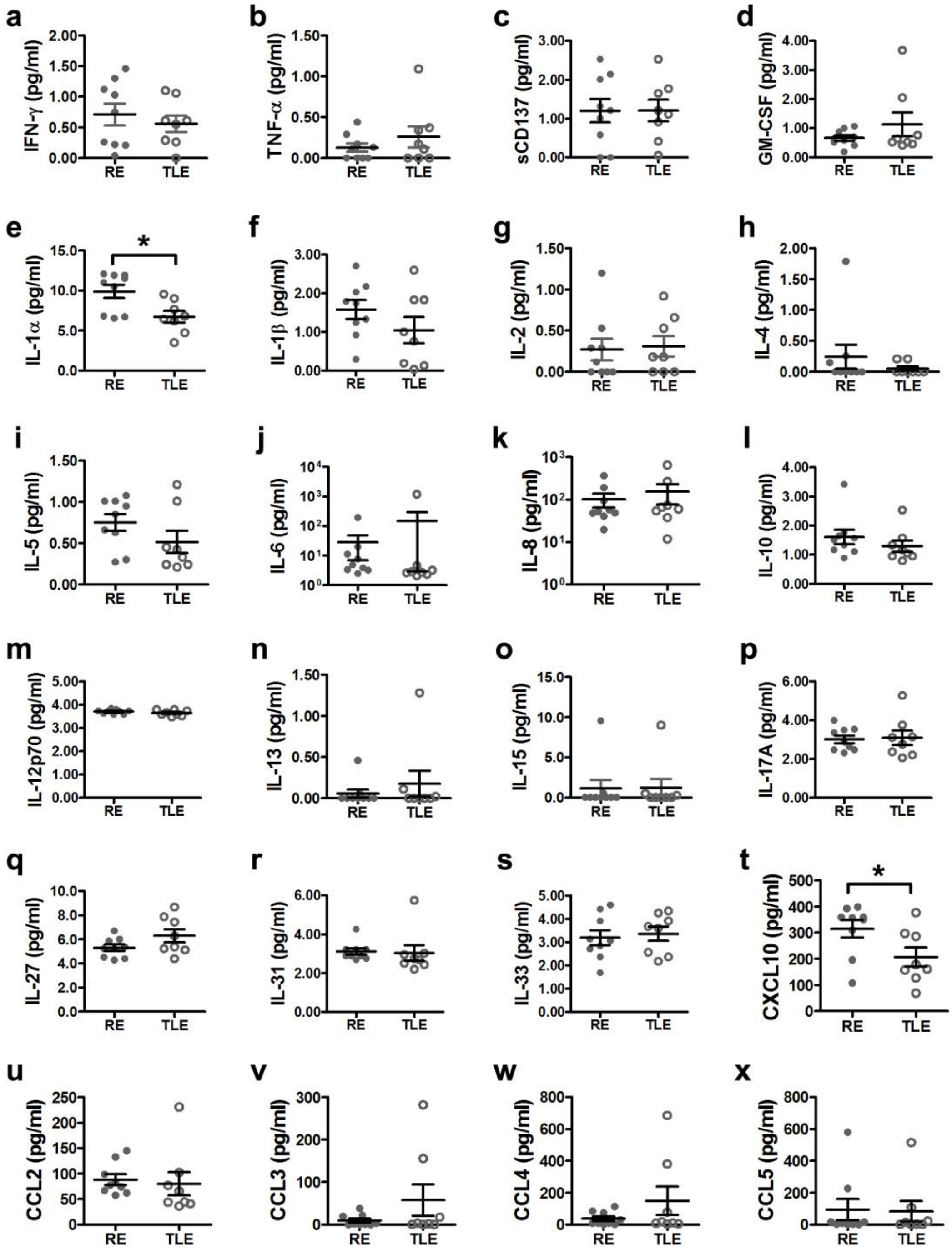

Supplementary Figure 6. The cytokine levels in RE and TLE cerebrospinal fluid (CSF). CSF from RE and TLE patients were analyzed using Aimplex Human Multiplex Immunoassays kits according to the manufacturer's instruction. The cytokine levels were expressed as mean ± std and analyzed with a two-tailed Student t test (n=9 for RE and n=8 for TLE). *: $p<0.05$.

Supplementary Table 1 List of antibodies

Antibodies	Manufacturer	Catalogue No.	Dilution
anti-HSV1	abcam, UK	ab9533	1:200
anti-HSV2	abcam, UK	ab9534	1:200
anti-VZV gB	santa cruz, USA	sc-56993	1:200
anti-EBNA2	abcam, UK	ab90543	1:200
anti-HCMV	abcam, UK	ab54023	1:200
anti-HHV6	abcam, UK	ab128404	1:200
anti-HHV8	abcam, UK	ab4103	1:200
anti-Iba1	abcam, UK	ab108539	1:200
anti-CD8a	abcam, UK	ab199016	1:200
anti-GZMB	abcam, UK	ab4059	1:200
anti-Iba1 antibody	Proteintech, USA	10904-1-AP	1:200
anti-TLR3	Abcam, UK	ab62566	1:200
anti-TLR9	abcam, UK	ab52967	1:200
anti-STING	abcam, UK	ab92605	1:200
anti-IFI16	abcam, UK	ab55328	1:200
anti-TBK1	abcam, UK	ab227182	1:200
anti-IRF3	abcam, UK)	ab218160	1:200
anti-mouse IgG H&L Alexa Fluor®488	abcam, UK	ab150117	1:1000
anti-rabbit IgG H&L Alexa Fluor®594	abcam, UK	ab150080	1:1000
